# What is in It for Them? Understanding the Impact of a ‘Support, Appreciate, Listen Team’ (SALT)-Based Suicide Prevention Peer Education Program on Peer Educators

**DOI:** 10.1007/s12310-018-9264-5

**Published:** 2018-04-06

**Authors:** Bobby Zachariah, Emma E. de Wit, Jyotsna Dnyaneshwar Bahirat, Joske F. G. Bunders-Aelen, Barbara J. Regeer

**Affiliations:** 1 Faculty of Earth and Life Sciences, Athena Institute for Research on Innovation and Communication in Health and Life Sciences, VU University, Amsterdam, de Boelelaan 1085, 1081 HV Amsterdam, The Netherlands; 2 Pune, India

**Keywords:** School intervention, Suicide, Mindfulness, Team, Peer education, Mental well-being, Adolescents, India, Peers

## Abstract

Youth suicide is a public health problem in India, and young people in school, particularly adolescents, experience heavy psychological burden. Prevention programs, involving peer educators (PEs), have proved useful strategies to address this problem, but their impact on the PEs is less understood, particularly in India. This qualitative study aims to explore the changes observed in PE students who were involved in a ‘mindfulness’ and ‘Support-Appreciate-Listen–Team’ (SALT)-based peer education program to address suicidal behavior in Indian school students. One hundred and fourteen students were trained as PEs in six high schools in Pune to identify and respond to the needs of students in distress. By listening to the narratives of the PEs, their parents, school authorities, and the associated NGO team, we reflect on perceived social, emotional, behavioral, and cognitive changes in PEs. The PEs demonstrated enhanced caring for those in distress both inside and outside school by improved listening skills, self-awareness, care, and empathy. Furthermore, the program had a positive impact on their broad emotional intelligence and PEs expressed increased ownership of life, taking action, and seeking support where needed. The study concludes that mindfulness and SALT-based peer education (PE) programs are valuable for the PEs. This could be used to motivate PEs to volunteer in such programs. Other results are discussed and further research areas are suggested.

## Introduction

Globally, suicide is the third main cause of death among youth (WHO, 2012), of whom many victims are in low- and middle-income countries. In India, suicides are the main cause of death among youth (Aaron et al., 2004), and the period from 2002 to 2011 has seen a 22% increase in suicide rates, of which about 34.6% are by young people (National Crime Records Bureau, 2013). In 2014, 1720 children below 14 years and 9230 below the age of 18 years committed suicide (NCRB, 2013–2014). According to other studies, the number of youth suicides is even higher. Patel et al. (2012) found that the percentage of youth suicide (15–29 years) was 56 and 40% among women and men, respectively, adding to a total of about 187,000 deaths in 2010. In a study conducted by Kharsati and Bhola (2015), it was found that more than 30% of the sample college students in India reported non-suicidal self-harming behavior in the preceding year.

This disconcerting pattern is often explained by the rapidly changing socioeconomic environment, with an increase in potential risk factors and a decline in protective factors in family structures, lifestyles, and social norms (Aggarwal & Berk, 2015). The rise of nuclear, dual-income families, combined with decreasing parental attention, is one of the negative outcomes of modernization often mentioned in relation to distress in youth (Arun & Chavan, 2009; Aggarwal & Berk, 2015; Bor, Dean, Najman, & Hayatbakhsh, 2014). In addition to the economic advantages these work opportunities bring, they also pose a risk of developing more materialistic family environments, and, as explained by Aggarwal and Berk (2015), can result in identity issues in adolescents and exacerbate the generation gap between parents and children, as traditional practices are replaced by modern lifestyles. In general, youth suicides, besides mental and physical illnesses, are often associated with increased pressures and competition at school, parental pressures, relationship problems, inter-generational conflicts, mental and or physical abuse, and fear of failure (Arun & Chavan, 2009; NCRB, 2013–2014).

Schools undoubtedly play a crucial role in influencing young people’s mental health. Within the school setting, youth seem to be both protected from as well as more exposed to risk factors for self-harming behavior (Patel, Flisher, Hetrick, & McGorry, 2007). School education equips students with skills that help them compete in the labor market, earn more money, and enjoy social stability (Radhakrishnan & Andrade, 2012). This embedding in a school setting may protect them from stressful life events (Patel et al., 2007), but school pupils, particularly adolescents, are also more vulnerable to experiencing psycho-social stress and self-inflicted harm (Arun & Chavan, 2009; Joshi, Gumashta, Kasturwar, & Deshpande, 2012). Besides academic pressures and fear of failing examinations, there are various risk factors connected to the school environment. It is often at school where children try out new forms of identity and social status in groups that can lead to emotional disturbance and maladaptive health behavior (Tung, Sandhu, & Singh, 2010), as well as violence and abuse (Patel et al., 2007). Adolescents undergo several important transitions, moving from one school to another, as well as from childhood to adulthood, encountering various new responsibilities and situations associated with this age (Hussain, Kumar, & Husain, 2008), while not necessarily possessing the skills to deal with these challenges. These stresses are exacerbated in more individualized, capitalist contexts, such as in the larger cities of India, where students are exposed to rapid globalization and the new value system it generates (Joshi et al., 2012; De Wit, Adithy, Bunders-Aelen, & Regeer, 2016). This is seen in the prevalence of other risky health behavior among Indian youth, such as unsafe sex and substance abuse, which could in part be explained as response to the conflicting messages from a globalizing world and the home environment (Tung et al., 2010).

Adolescence is furthermore a period where parent–child relations are actively restructured and peers become more important for the person’s development (Moretti & Peled, 2004). Adolescents progressively use horizontal relations with peers to explore their self-value and find out who they are. Social networks and peer affiliations are forged in the school setting and play a pivotal role in determining the mental and emotional well-being of those involved. Social exclusion and bullying are a frequent and ugly flip-side of these social networks, and a major cause of stress and anxiety among many adolescents (Fekkes, Pijpers, Fredriks, Vogels, & Verloove-Vanhorick, 2006). Whether positively or negatively, peers play an important role in the lives of adolescents, and nurturing and positive relationships among them could undeniably function as means to protect against various life stressors (Capuzzi & Gross, 2014). The World Health Organization (WHO)-based collaboration for research on health behavior in School-aged Children (HBSC) therefore reports that: *‘Peers are valuable social contacts that contribute to young people’s health and well*-*being’* (HBSC, 2010, p. 7) and that *‘family, peers and schools can provide social supportive environments for healthy development’.* Other studies show that students in distress often first seek out their peers for help, and suicidal students often tell a friend or other school students about their anxieties and suicidal ideas (Drum, Brownson, Denmark, & Smith, 2009; Eskin, 2013; Kalafat & Elias, 1994).

### School-Based Suicide Prevention Programs

Adolescents spend a significant amount of time with their peers at school or in school-related settings (Garofalo, Siegel, & Laub, 1987), and this environment plays a considerable role in shaping their (mental) health. Schools have the infrastructure to reach students, and the credibility and trust of parents to introduce learning programs (Latha & Reddy, 2007). Patel et al. (2007) therefore argue that schools provide a unique opportunity for the early identification and reduction in suicide risk factors. School-based suicide prevention programs are also widely recommended by various other researchers (Gutierrez, Watkins, & Collura, 2004; Mazza, 1997; Eckert, Miller, Riley-Tillman, & DuPaul, 2006; Reynolds, 1991; Shaffer & Craft, 1999). There is strong evidence that peer education can help to build awareness of warning signs of suicide and training of life skills which in turn can lead to greater self-efficacy, problem-solving skills, and self-reported vulnerability to suicide (Miller, Eckert, & Mazza, 2009). Furthermore, suicide prevention programs have proven much more effective when carried out by fellow students. As Wyman et al. (2008) note, based on their random control trial (RCT), fewer than 20% of 8th and 10th graders who have recently attempted suicide indicated they would talk to a counselor or another adult at school if they needed help. PE programs,^1^ which empower students to respond to peers in distress, are internationally used in suicide prevention and show promising results (Guo, Harstall, Collins, & Dennett, 2006). Kalafat and Elias (1994), based on their research in Israel, note that PE programs can increase suicide awareness in the population and increase the likelihood of suicidal students to be identified in time. Orbach and Bar-Joseph (1993) detected a reduction in suicidal inclinations in four high schools, after implementing PE, particularly among young women. Finally, a study conducted at 18 US high schools shows that PE programs can increase suicide awareness and students’ inclination to seek help from adults (Wyman et al., 2010). Overall, these studies as well as the endorsement of international initiatives such as UNAIDS (Kerrigan et al., 1999) show the relevance of school-based PE programs to combat issues such as suicide and distress in youth.

An important but less well-understood question is how these programs benefit the large and growing group of voluntary student peer educators (PEs). Any PE program first and foremost focuses on the recruitment, careful training, and continuous monitoring of PEs, and their psycho-social development in case of suicide prevention programs (Ebreo, Feist-Price, Siewe, & Zimmerman, 2002). Indeed, most PE teams are set up to teach PEs relevant life skills, such as being attentive and compassionate to others and to oneself, to learn to initiate action and seek help, which in themselves contribute to a person’s development (Drum et al., 2009; Ilakkuvan, Snyder, & Wiggins, 2015; Wyman et al., 2010). Some studies have shown that PE programs can bring about positive outcomes in the lives of PEs, such as improved academic performance, ability to solve schoolwork problems, enhanced social development (Topping & Whiteley, 1993; Vijayakumar, 2004), better communication or social skills (Pfeffer, 2001), and enhanced self-esteem (Vazquez, 1990 as cited by Ebreo et al., 2002). PE programs in various fields also boost the PEs’ social status due to their new role (Vazquez, 1990; Ebreo et al., 2002). In the specific field of suicide prevention, a US study shows that PE programs can positively change PEs’ attitudes toward suicide, reduce stigma, improve their relationships with other students and their engagement in schoolwork (Wyman et al., 2010), and improve their social, emotional, cognitive, behavioral, and attitudinal development. How a PE program contributes to friendships and other relationships (social development), self-esteem or sense of self-identity (emotional development), cognitive development, and/or ways of coping with schoolwork, drugs, and tensions (behavioral development) needs further exploration. In the field of suicide prevention in particular, the impact on PEs has been insufficiently studied. Particularly, in the context of quickly modernizing contexts, the implementation of PE is promising, as it is tailored to the increased need for safety, self-efficacy, and social connection among peers (Zhao, Selman, & Haste, 2015).

### Objective of the Study

The current study aims to explore the impact of PE programs on the social, emotional, cognitive, behavioral, and attitudinal changes among voluntary PEs in the context of suicide prevention in India. We were interested in understanding the changes they underwent, with the aim to encourage and strengthen PE approaches in the future.

## Peer Education: Theoretical Background and Current Intervention

The working mechanisms of PE programs, including the training and monitoring of PEs, are well established in the literature. These programs have been implemented worldwide since the 1960s in diverse forms for a variety of objectives, and particularly in the last few decades, to address mental well-being in school children (Weare, 2015). The notion of peer education is reinforced by many social theories (Catanzarite & Robinson, 2013), including the social learning theory of Bandura (1977) which argues that when students are given accurate training and encouragement, they can successfully induce positive change in lives of their peers (Catanzarite & Robinson, 2013). Freire’s theory of participatory education (Freire, 1970) is another important theory on which PE is grounded, with its focus on the empowerment of people to participate in learning and social change. Peer education is, in this approach, grounded in the observation that many people make changes not based on what they know, but on the opinions and actions of close and trusted peers (Kerrigan et al., 1999). Finally, peer education is, as Drum et al. (2009) proposes, also a ‘population-focused paradigm’ for suicide prevention, in which suicidal behavior and its ‘submerged elements’ are perceived as inherent in many features of the school context, which therefore need to be shared and addressed by the whole school community. He describes how the population-focused approach of PE, as opposed to individual-focused mental health approaches, can do more to *‘capitalize on the countless opportunities both to prevent development of the symptom (suicidal behaviour) and to reduce forces routinely contributing to life*-*threatening intensification of suicidal intend’* (Drum et al., 2009, p. 220). Therefore, as Ilakkuvan et al. (2015) point out, the challenge is not to determine *whether* peers should be involved in strategies to prevent suicide (they should because they are part of the school environment that is potentially and inherently contributing to distress in individual students), but rather *how* they can be effectively involved.

Definitions of peer education vary by program and the specific topic it addresses. PE programs can range from informal to more professional structures, but the main focus is to use the interactions between peers as vehicles to delineate problems, encourage help-seeking behavior, and provide a listening space and, if needed, professional support to peers (Ilakkuvan et al., 2015). For most PE programs, having a clear vision, as well as training, supervision, and support, is an essential guiding element. Training should set out clear cognitive, affective, and behavioral outcomes concerning the PEs’ learning goals.

In the context of suicide prevention (Capuzzi & Gross, 2014; Catanzarite & Robinson, 2013; Ilakkuvan et al., 2015; Taub & Robertson, 2013), such outcomes generally involve increased knowledge of suicide warning signs, skills to respond to peers in distress, empathy and active listening skills, increased communication and/or presentational skills, an understanding of their role and its boundaries (e.g., awareness of the limits of their aid and knowing when to consult a professional), non-judgmental thinking about others, increased self-care, and an awareness of when to protect their own well-being. ‘Attentiveness to students in distress,’ ‘empathic listening skills,’ ‘leadership qualities,’ and ‘seeking self-assistance’ are key elements of a successful PE training (UNODC, 2006). Such guidelines also stress that training for PE educators or helpers should be facilitated by professional mental health workers (including psychologists and social workers) to ensure that life skills are taught effectively and the well-being of PE helpers is monitored. Such considerations are imperative to ensure that PEs themselves are in a good mental state and capable of responding skillfully to the needs of others in distress (Capuzzi & Gross, 2014), thus underlining the relevance of this study’s main question with regard to the development of PEs.

### Peer Education in Pune

The basic philosophy supporting the working mechanisms of the PE programs in this study is the ‘Human Capacity for Response’ (HCfR), also referred to as the *‘Community Life Competence (CLC)’* Framework (Community Life Competence Approach, 2017). The HCfR framework reflects the belief that people have the capacity to *care, change, hope, lead and belong to a community* (such as a school community in our case), and that communities can harness these capacities to collectively address challenges (Lamboray, 2016). Globally, the HCfR approach has been used to address a wide variety of issues including HIV (Campbell & Rader, 1995), malaria (Malaria Control Tools—A Midterm Evaluation of the Malaria Community Competence Process in Nine African Countries, 2009), polio (Chu & Norman, 2014), and psycho-social support for communities after disasters (Hosford, 2006). As a theory, the HCfR is strongly rooted in the *Strengths*-*Based Approach* commonly practiced and recognized in social work. The strength-based philosophy essentially celebrates the resources, resilience, and agency of individual people and communities (Rapp & Sullivan, 2006). It builds on the positive psychology of hope and optimism regarding the ability of people and situations to make better choices and transform, particularly when various actors work together (Rapp & Sullivan, 2006; Gray, 2011). As such, the notion of connectedness is also deeply embedded in strength-based approaches, which involves a sense of belonging, caring for and valuing each other, and mutual trust, as well as a sense of responsibility and following positive norms and expectations (Tingey et al., 2016). Furthermore, it has a future-oriented focus, empowering communities to establish clear goals and visions to achieve improvement. The most distinguishing element of the strength-based approach is the notion that persons are not problems that require a distance between ‘helper’ and those who receive help. In that sense, it moves away from the deficit model and emphasizes strengths and people’s ability to help themselves as well as each other (Gray, 2011).

The key approach that expresses the HCfR approach is Support, Appreciate, Listen and Team (SALT), Dream Building, Self-Assessment, Action Planning and Self-Measurement of change (Morea, Kamasua, Zachariah, & Kampe, 2009). The SALT approach and dream building were adopted in the peer education program module. ‘Active listening’ and ‘mindfulness’ of emotions were a dual strategy used by ‘Connecting…NGO’ in the training of suicide prevention helpline volunteers. Active listening helps distressed callers to feel acknowledged and their feelings validated, thus promoting healing. ‘Active listening’ was considered as a key strategy for change in training peer educators. Mindfulness (Kabat-Zinn, 2003) was practiced as a healing tool by helpline volunteers while listening to a person in distress. Mindfulness is focused on non-judgmental observation of current experiences, by attending to the breath and sensations of the body. Based on the helpline experience, it was hypothesized that mindfulness-based exercises would have various positive effects, such as increased emotional regulation and awareness and heightened concentration levels (Chesin & Jeglic, 2016). Mindfulness was thus included as a key element of the PE curriculum. Emotional Freedom Technique (EFT) (Scott, 2008), also called tapping or meridian stimulation, was taught to helpline volunteer listeners to promote emotional healing. EFT was included as a practice for PEs to reduce feelings of anxiety and stress (Church, De Asis, & Brooks, 2012).

With regard to the actual intervention, this PE program is focused on creating an effective network of volunteers who are compassionately engaged with their direct environment, mainly their school peers. This means that the program follows a cycle of (1) recruiting PE volunteers; (2) training PE volunteers; and (3) continuous mentoring of volunteers and reflecting on their goals and ‘work’ as volunteers. This process is guided by professional psychologists and trained adult volunteers. Throughout the year, PEs come together weekly under the guidance of adult volunteers in their own school to learn, openly share, and support each other. PEs are encouraged to share their experiences and support each other in recognizing and responding to distress in others. The key elements of the PE curriculum include: (1) theoretical framework for PE program—Human capacity for response and Community life competence program, (2) Role of a Peer Educator, (3) Self-Care (Mindfulness and EFT), (4) Active Listening, (5) Understanding Suicide, and (6) Ways to Respond. Further details of the curriculum are provided in Table 1 of “Appendix”.

**Table 1:** Peer educators program training module

Module 1	Theoretical framework for PE program Human capacity for response/community life competence approach Key beliefs Community capacity to care, change, hope, leadership, and be a community Key approach Support—appreciate—listen—learn
Module 2	Role of a peer educator Expanding spheres of influence Listening Working as a team Discerning when to seek help of trained adults
Module 3	Self-care Mindfulness Emotional freedom technique
Module 4	Active listening Good listening and bad listening (micro-skills and macro-skills of listening) Listening without judgment Acknowledging emotions
Module 5	Distress and ways to respond Levels of distress Understanding thoughts—emotions—body reactions Supporting the person in distress Handling high distress and suicidal feelings. Understanding shared confidentiality—involving responsible adults

## Methodology

### Research Context and Recruitment Process

This study was implemented in six high schools in the municipality of Pune, a large city in Maharashtra state with a population of about 3 million, of whom more than 0.5 million are in the 10–15 age group (Census Data, 2011). Pune is a national education hub because of its abundance of academic institutions and foreign students. This situation leads to high levels of competition and stress among youth. There is a need for interventions to prevent and address mental health problems.

Connecting…NGO,^1^ a volunteer-based organization dedicated to suicide prevention and survivor support, is the implementing organization for the PE programs. After various discussions with school authorities to address the concerns about suicidal ideation, self-harming behavior and emotional distress among students, Connecting commenced the PE program in 2012 in eight schools. Two schools subsequently left the program, due to difficulty in allocating time in the curriculum. Students involved as PEs were aged between 12 and 15 years. Most of them were from middle-class or upper-class families, with parents’ occupations ranging from government officials, small-to-large-scale business owners, educators, lawyers, etc. Most parents had pursued higher education.

For the recruitment of PE volunteers, information was sent out to students and parents, inviting them to a presentation, which was held in the schools. Letters of consent were obtained from each participating PE volunteer and their parents. Meanwhile, voluntary adult facilitators were trained to mentor the peer educators in the topics covered by the curriculum in a 4-day training session at Connecting, before being assigned to a participating high school. Weekly peer education training sessions were held inside the school premises by trained facilitators.

Subsequently, over the course of 8 months each year, weekly meetings of 45 min (details in Table 2 in “Appendix”) were organized in the school environment and during school hours. The latter was required to make it easier for volunteers to join and facilitate high retention numbers. During sessions, PEs were encouraged to share their experiences of reaching out to those in emotional distress. These experiences were processed in the group to facilitate joint learning.

**Table 2:** Cross section of a weekly PE session facilitated by adult program facilitators (45 min)

Mindfulness session—focusing on here and now	5 min
How did we work as a peer educator in the past week? Experience analysis and lessons learned	15 min
Learning from peer education program module	20 min
Mindfulness session—listening to our emotions and being present	5 min

### Methods

The questions guiding this research were focused on understanding the emotional, behavioral, cognitive, and social changes the PE volunteers underwent by participating in the PE program for at least one school year (which includes a complete cycle of PE training). For the study, we relied on mixed qualitative data collection methods, including focus groups, semi-structured interviews, and open questionnaires (Creswell, Shope, Clark, & Green, 2006). A variety of sources were included to develop answers to the proposed questions, including parents, school authorities, the PEs, and Connecting volunteers (see Table 3 in “Appendix”). Questions posed directly to the PEs were administered through open questionnaires, and included items, such as: ‘*How was your overall experience as participant in the PE program? What personal changes did you experience as result of the program? What parts of the program impacted you mostly? How does this make you feel? How did you learn to reach out to peers? What would you still like to learn? and, How could the PE program be improved?* Questions posed to others about the development of the PEs were used to support their reflection, such as: ‘*What are your perspectives on the value of the PE program for students? What changes have you witnessed in your pupil/child as result of their participation in the PE program? How do these developments influence others in the school or outside the school?*

**Table 3:** Summary of data sources and methodology

Data collection method and source of data	Key questions explored
*Questionnaire completed by peer educators* 76 (20 girls and 56 boys) out 114 peer educators from 6 schools completed the questionnaire Peer educators are 12–13 years of age and participated a minimum of 1 year in the program	What changes have you observed in yourself as a result of participation in PEP? How do you feel this program has helped you? How many people have you reached out to since your participation in the PEP? Please share about one or more times when you reached out to someone which had the most impact on your learning experience. How did it make you feel?
*Focus group discussion with parents of peer educators* Parents are 35–45 years of age and belong to middle-class families	What is your opinion about distress among students? What is your opinion about the intention/impact/quality of the PEP? What changes have you noticed among your children who volunteer in the Peer Education Program? What changes have you noticed in the skills, attitudes, and behavior of the peer educators?
*Interview schedule with one school head master and two school counselors*	What are your views about distress among school students and suicide and prevention? How has the peer education program helped the peer educators? What changes have you noticed among them? What recommendations do you have for further improvement of the program?
*Peer education programme session reports* (152 reports) gathered over a period of 2 years Online forms filled by PEP facilitators after each session. Combined data accessed by authors from central data base at the organization	Name of person filling in this form: How many connecting volunteers were there? Name of volunteers: School name: Date of event This activity was conducted with: (parents, teachers, peer educators, etc.) Number of students: Was there anybody else present at the event? What activities/discussions happened during the session? What concerns were discussed? What conclusions were reached? & what follow-up is required? Interesting human story demonstrating concerns and capacity of people: Facilitators feedback regarding the session

A careful data triangulation approach was adopted to make sense of the data that was gathered (Mathison, 1988). Responses to questions were compared and adopted as valid when they were brought up or confirmed by at least one other actor (Webb, Campbell, Schwartz, & Sechrest, 1966).

First, PEs who were involved with Connecting for at least one school year were invited to fill in an open-ended questionnaire with topics that helped them report on self-observed changes that they believed had resulted from participating in the PE program. Students were asked to elaborate on the influence of PE participation on their development in relevant fields (Seiffge-Krenke, Aunola, & Nurmi, 2009), including *school, future, parents, leisure time, peers, romantic relationships* and *self*-*related concerns*. Second, focus group sessions were organized with 24 parents in total to reflect upon the changes they had observed in their children after having participated in the PE program. The focus groups lasted approximately 2 h and were facilitated by the first author and assisted by two Connecting volunteers. In addition, semi-structured interviews of approximately 1 h were conducted with school authorities to reflect upon the changes observed in PE volunteers, and compared to students who did not participate in the PE program. Similarly, semi-structured interviews were conducted with 10 PEs (six girls and four boys) who were involved in the program for 2 years, as well as four program facilitators (two men and two women). Questions were raised regarding the impact of PE program on the lives of PEs. These included the reason for volunteering, their role as a PE, their emotional state as they listened to the distress of others, methods of dealing with own emotional distress, and self-reported changes. Finally, the content and reflections of PEs and facilitators were documented for all PE session using the online documentation tool Formstack (www.formstack.com), which resulted in a total of 152 session reports over the course of two years. The documentation included details of the performed activities, lessons learned by PEs, and their experiences with the program so far.

### Data Analysis

This study was explorative and inductive. Hence, we used an open and axial coding approach (Strauss & Corbin, 1998). The audio tapes of focus groups and interviews were transcribed verbatim. The answers to the questionnaires were digitized. All data were imported into MaxQDA software for analysis. The data from PEs’ questionnaire were analyzed by the first and second author independently by comparing answers to questions and abstracting distinct themes until no new themes could be discovered (Grove, Burns, & Gray, 2015). This provided a preliminary idea of what influences the program had on the PEs, both positive and negative, and which categories might be useful to analyze the data. Subsequently, we looked for patterns and categories in the rest of the data sources to specify these categories further, make more distinctions if necessary, find properties, dimensions and observe relations between the various categories (Strauss & Corbin, 1998). For instance, some distinctions were made between these self-reports of students, and whether these were substantiated by other sources of information. Subsequently, items that were not mentioned by all participants were either not included in the results or described more cautiously. Axial coding was then used to further distinguish categories, particularly with regard to emotional, behavioral, cognitive, and social dimensions, and the interactions occurring within these categories (Grove et al., 2015). The resulting categories were cross-checked by the third and fourth authors, and final refinements were made to distil the results from the analysis.

## Results

In this section, we describe the changes of a total of 76 PEs (20 girls and 56 boys) who completed the questionnaire, which is about 67% of all 114 PEs who were involved in the program between 2012 and 2015. The results in this section are divided into two parts. First, themes which are related to the expected outcomes of the program, also known as ‘direct effects’ in the social support literature, will be described, Second, the themes which were less anticipated, or ‘indirect effects’ of the PEP, will be discussed. Together, recognizing the complex direct and indirect themes that emerged from the analysis, the implications of these effects on the unique challenges and opportunities for peer leaders to grow and develop new skills, are considered within the context of future PE models for suicide prevention in India.

### Direct Effects of PEP Program

#### Caring for Others

In this section, we briefly report the changes that were noticed in PEs and were expected outcomes from their training. Overall, what came out from the analysis was the increased self-reported motivation and ability of PEs to care for others, as well as the development of skills that this requires. The themes that emerged are the PEs’ greater ability to (1) recognize distress in others; (2) reach out to people in distress; (3) use listening skills and non-judgmental approaches; and 4) conduct the activities as a team. These skills were imparted in the PE sessions in the schools.

#### Recognizing Distress in Others

The capacity to detect and understand emotions (particularly distress) in other people was enhanced according to PE volunteers themselves. This is witnessed, for instance, in the statement of a male student:

I noticed that a friend looked sad and not attentive. I decided to just ask him what was wrong. He told me his situation. He was feeling very low because all his old friends were no more in contact or left because they had a fight. And he was confused about his life.

The mother of a PE spoke about how her daughter supported a neighbor who was dealing with distress and addictions, saying: ‘*My daughter would tell me: “I am not supposed to reveal anything, but trust me, I need to be there with her you don’t need to be around”. As parents, we always wonder, “I hope that this is ok, that she is not getting into any trouble”, but then I eventually learned to trust her with this’.*

School authorities also mentioned that one of the key lessons PE’s learn is how to share their feelings and that they were better able to analyze and discuss their emotions and behaviors in various settings.

#### Reaching Out to Others

Besides becoming more aware, PEs also reported being more confident in their ability to reach out to others and respond to their needs. Most students felt they had had an opportunity to practice SALT in their response to others, which included a variety of people who were experiencing different levels of distress. Among them were 10–15 people in medium distress and six who had made plans to commit suicide. The means used by the PEs to support these people included listening and accompaniment, and sometimes advising, until the risk was alleviated. A female PE shared her experience:

One of my friends was in such distress that she was contemplating suicide because she was thinking that she is worse than others. I noticed this and listened to her. She cried and told me what her problem was and why she was thinking of killing herself. I then dealt with her situation using the SALT approach, explained her to trust herself and have guts to confront life.

The School Counselor explained how PE students learn to be better able to reach out to others:

When they learn about themselves, that is when they become better at understanding the emotional stage of someone else. So, when they focus on others, it’s like they can draw a common wavelength between them and peers, even if they deal with different situations. They can connect with their emotional status. So, the more PEs understand their own emotions and thoughts, they are able to tune into the emotions of others, whether its peers, parents or other significant people in their lives.

Not all students have applied these lessons, however. Some PEs expressed their hope to eventually reach out, though they did not feel confident to do so yet now. The reasons attributed include ‘not yet found anyone in distress’ and ‘not feeling confident of reaching out.’

#### Application of Listening Skills and Non-judgmental Attitude

The PEs used a variety of approaches in reaching out to their peers. A higher-order skill the program aims to impart is to express care by active listening and not interrupting with suggestions and advice. About half the students reported their ability to listen and practice their active listening skills, by probing, summarizing, appreciating, and supporting those in distress. The reports of students indicate a significant change in this aspect as result of their participation in the PE program. One male student reported:

Patience is another thing I learned. I was used to that if someone discussed some issue of problem, that people would start judging that person; this even happened in 11^th^ standard. But peer volunteers, even those who would be in 8^th^ or 9^th^ standard, would already be known for being non-judgmental and people would come for telling their stories. We learned also to first listen, before advising (only if advice was invited). So, the level of maturity and understanding has changed in me.

Equally, parents reported positive changes in their children. A mother reported how her child was applying the learning at home:

She is a very good listener. Now if there is anything, like there is an issue between me and my son, she listens to me separately and my son separately. It helps us to understand that there is nothing to it as we had thought. This is how she helps us calm down and solve issues.

On the other hand, there were several examples in which students showed eagerness to provide advice and find solutions, as is also witnessed in some of the stories displayed earlier. For instance, a female student mentioned:

My friend was feeling bad because her family had problems, and I explained to her that everyone had problems and she could just overcome it.

#### Team Work and Bonding

The PE program itself provided a listening space where the PEs could trust each other to share both their personal and their PE volunteering experiences, be supported and learn together. During these sessions, it was noticed that PEs themselves also experience various moments of distress due to academic and parental pressures, conflicts in their social and romantic life, or issues related to their self-identity. Through the weekly sessions with the whole group, students learned to discuss issues that they were concerned about. The students also contacted each other for personal issues, for instance, as seen by a student who said, for instance:

I was feeling very distressed because of my brother. He didn’t trust me anymore and the relationship was not good. For the solution, I talked to the other PEs in my school. They supported me in handling the problem and build my trust back with my brother.

PEs also shared that the PE program provided them with a social group that helped them stay truer to themselves within the wider school context of peer pressure to engage in drinking, smoking, or other things they did not feel like doing. One PE said, instance: *‘I had friends who were drinking and bunk classes, and not doing these things made me feel left out. The PE group, however, made me feel that I can say NO to such things, and I will never really be without friends’.*

Counselors also reported development of belongingness and a sense of association in the PEs:

Among the PEs, when they are sharing something, they realize what is being shared is similar to what they are going through and they are able to receive wisdom by hearing the analysis of the situation. They feel that they are not the only one who is rejected and broken at times. Being in a group, they related and belonged to each other. This belongingness helps them – same age group, similar kind of issues.

A program facilitator shared how the PEs cultivate team building: *‘In beginning the group was small. However older PEs encouraged new students to join, so the group grew a lot’.*

### Indirect and Unanticipated Results of PEP Program

In the previous section, we briefly reported the changes noticed in PEs that were expected from the training they received. In the following, we will reflect on two changes that were reported by various actors, which show a secondary learning effect that PEs used in broader life settings. First, we reflect upon the increased emotional awareness of PEs and the way they have matured in their emotional intelligence. Second, the increased independence in PEs and their sense of agency is described.

#### Emotional Intelligence

What became very evident from the responses of parents, PEs, as well as counselors is that, first, the *emotional awareness* of PEs has grown. This includes the ability of students to *introspect* and patiently explore which emotions and sensations they experience. One PE said about this:

I had a lot of anger issues in my pre-training, but after the training, I have calmed down a lot, the connecting NGO’s tricks have helped me to become aware of what the current feeling is, and in trying to find out the root cause of why I feel what I feel. My patience has developed in me immensely.

A school counselor explained how the program helped the PEs to recognize their emotions and work toward solving them. She shared the example of a PE who lost her sister to suicide:

She had got into a conflict mode with people around her. Due to PE program, some of her conflicts were settled at that time. This has happened due to her acceptance of her issues with herself and some of her friends at that time. She learned to maintain relationships with friends. And then there was a valuable change in her.

Second, there was an increased sense of *mindfulness* in students, which positively surprised parents, who repeatedly mentioned their child’s relatively rapid emotional maturity, and how a more accepting, understanding attitude also improves their relationships. Parents particularly noticed a difference in how much their children started sharing with them, which is an interesting phenomenon, as adolescents usually tend to disclose less to their parents at this stage of their development. This had to do with the increased ability of the PEs to understand their own emotions, as well as those of their parents. One parent explained, for instance:

My daughter has changed a lot, particularly in her talking to me. She’s become polite, she tries her best to understand me. She understands now that I am a doctor and I cannot give her the same amount of time as other mothers give to their children. She complains less about this now. And because she shares her own thoughts more, we have become better friends.

Third, counselors also reflect on the students’ *emotional development* and how they have grown. One counselor explained:

Because they need to relate to the emotions of their peers, they have started to develop an awareness of their own emotions. We could see this development happening from session to session, as their emotional maturity was reflected in their discussions. It really empowered them to look at their own feelings, to analyse them. They do self-inquiry on why they are stressed, which they wouldn’t have done if they weren’t first aware of their emotions.

Similarly, and finally, we saw the additional benefit (more than a skill) of an increased feeling of *self*-*worth* as well as *self*-*care*. A Connecting facilitator remarked how this has helped in building confidence and changed the way they are building friendships:

For many PEs, their involvement in the program has changed the way they think about their own worth. This is reflected in their relationships with their peers. They now try to find new friends, who are more trustworthy and caring about their secrets.

They start realizing the value of human beings, particularly the value of their own life and how they live. Several students reflected on this, explaining how through the PE program they have become self-reflective. Students said things like: ‘*I have solved the problems of my thoughts, which changed my behavior’,* or ´*I noticed that I have different feelings in different situations’,* and *‘it helped me to take away stress and tensions’.*

This has also helped the PEs in being noticed for their leadership abilities, which in some cases led them to become class representative or take up other leadership functions in the school.

Overall, the increased emotional awareness is particularly witnessed in the way PE students bond with others, but also how they handle the relationship with themselves. As such, counselors noticed that students that were part of the PE program also feel more secure and self-reliant. Another counselor mentioned:

The self-confidence of these students has been built in the months that they were participating, as well as their ability to deal with their stress. For instance, there was a student with quite severe anger issues, which she resolved mostly after joining the PE program by having a constant ability to share what was happening with her.

#### Increased Ownership and Agency

For many students, the involvement in the PE program has meant a change in how they reflect on themselves and the world around them. They have become more independent, more critical, and active in their approach to change negative aspects of their context. This is reflected in most of the actors. Parents, for instance, explained that after joining the PE program, their children have become more confident in speaking to people, in seeking knowledge and understanding, and taking decisions, sometimes even beyond their parents’ expectations. PEs have taken up several issues as their responsibility to confront, including conflicts between their own parents, conflicts between neighboring parents and their children, conflicts between siblings, and supporting peers who were being bullied. To do this, the PEs have used a combination of skills to find a solution, including noticing distress, approaching people, listening to them, negotiating, and involving others to find a solution.

When we asked PEs how the PE program has changed them, they also show increased sense of ownership and agency. One student expressed about her development:

I have a cousin of mine, who liked a guy and wanted to marry him but her parents were against the marriage and were forcing her to marry a boy of their choice. She talked to me about this matter and I decided to talk to their parents. In an understanding and polished way, I explained the situation to them. And at first, they were against it, but then they were ready to change their position. And the girl got married to the boy that she wanted to be with. And this was the biggest achievement of my life! It was a great feeling to achieve something like this.

The parents also mentioned that their children’s academic approach has changed, as they have become more independent. They are willing to seek help when needed. And they want to transmit the things they learn to others. Finally, the PE program has opened their eyes to social injustice and the will to do something about it. A parent shared, for instance:

I am very happy that my daughter wants to do good to the society. I will refer to an incidence. A friend of my daughter (though this is not a really close friend), approached my daughter and confessed about her family’s domestic violence. As a mother, I felt that my daughter was definitely not mature enough to deal with this matter. But, I was wrong! She stood as a pillar of support to this family. She supported her, she boosted her friend’s confidence and she stood by her so that it did not affect her schooling. With good supportive counselling that girl is in a good school now, regardless of her parent’s marital separation. I am proud of my daughter, who I thought way too young to respond to such a situation.

Another parent reflected on her daughter’s development as result of the PE program in terms of ‘*coming into existence*’ and *‘becoming an individual’.* She said that her daughter for a long time seemed to not mature or grow into her own being. It was after she participated in the PE program that she started becoming more honest, open, and able to speak up for herself and others.

The teachers who teach the students also notice changes among the PEs. One teacher shared her experience as follows:

I notice lots of changes among the PEs. Whenever I am teaching, the students tend to just look down and don’t interact with me much. But over time the PEs have changed. They have started thinking more critically and asking more questions. They are looking at me and interacting more.

Finally, a PE reported on the ownership of their life vision and hence ability to take action as needed and get back on track when they realize that they are straying:

My academics improved a lot due to the PE program. And being a peer educator, when you understand what your aims are, because you are able to say no to things, take a stand, take a right decision, filter out the other things … we are able to focus on what matters and you are able to focus better in academics. I lost my dad when I was in 8th grade as he died by suicide. First, I got diverted and received less marks, but then I managed to get myself back on track and see what is important for me and my family.

## Discussion

This study was conducted to explore the potential impact of a PE program on PEs involved in a school suicide prevention program for at least one school year. To our understanding, the impact of such a program on the PEs themselves remained understudied in the context of India and in the academic literature, which motivated this current study.

We first found that participation in the PE program had an impact on four distinct areas of skills. PE volunteers developed supportive attitudes and skills in the areas of identifying distress in people, reaching out to those in distress, supporting them by active non-judgmental listening, and improving the teamwork and bonding among PEs. We considered these skills as first-order or single-loop learning, as these skills were necessary to respond to distress in the existing setting, without necessarily challenging the norms of the system (Argyris & Schon, 1974; Watzlawick, Weakland, & Fisch, 1974). Argyris and Schon (1974) explain that these single-loop learning skills are a necessary means to serve an established end. This means that through single-loop learning, the learner is able to use skills in pre-imagined situations and for a particular outcome. The envisaged objective in this case would be to prevent suicidal ideation/distress/negative emotions in peer students, using the SALT approach. These lessons were expected, and we see that PEs were able to use these required skills in recognizable settings, such as a distressed student in the classroom or an emotionally disturbed friend. Still, with regard to PEs’ listening skills, students varied in their ability to postpone judgment or give advice, as we could see from their self-reported stories which involved giving some form of support to another person. Some students would perhaps make a suggestion or propose a solution to the distressed person a bit too soon. We know that, although taking the person in distress seriously and actively supporting him or her in finding better ways to cope can be useful, giving advice too soon can lead the person to close down and feel misunderstood (UNODC, 2006). The skills of active listening, asking clarifying questions, and showing non-judgmental concern, however, are not easily acquired, even with training, and require therefore continuous practice (Cross, Matthieu, Cerel, & Knox, 2007).

Second, it was interesting to note that the PE program had an impact on the PEs’ emotional development by way of increased emotional awareness and patience, feelings of self-worth, practice of self-care and building friendships, which were deeply embedded learning benefits, which could also be applied in other settings. We refer to these lessons as double-loop or second-order learning, described by Argyris and Schon (1974) as: *‘Not merely seeking out alternative actions to achieve the same ends, but also the appropriateness and propriety of these ends’* (cited in Greenwood, Model, Rydell, & Chiesa, 1998, p. 1043). In this sense, using the SALT approach to prevent suicide was expanded by the students in their critical reflection on their own well-being and that of their broader surroundings, and in making active attempts to change this—lessons that they can apply in various contexts throughout their life. For instance, since participating in the program, PEs had also become more open to sharing their concerns with their parents, resulting in better communication, opportunities for dialogue and understanding, and improved relationships at home.

These results are similar to those exhibited by an HIV PE program where the PEs became more open to sharing their risk behaviors with their parents (Ebreo et al., 2002; Pearlman, Camberg, Wallace, Symons, & Finison, 2002). Allen, Philliber, and Hoggson (1990) argue that participation in PE programs may shape youths’ pro-social competence and reduce problematic behavior. The growing independence displayed in reduced dependence on parents and teachers in dealing with their own issues are relevant indicators. These results are somewhat similar to the outcomes from a HIV PE program (Strange et al.), and an HIV, sexual health, drugs evaluation project (Backett-Milburn & Wilson, 2000), which reported that the PEs had experienced ‘increase in self-awareness’ (Hamilton & Fenzel, 1988), greater confidence, were able to ‘understand both sides of the argument’, ‘able to express thoughts and opinion’ and ‘not shy of expressing what they want’ from a relationship. All these are important life skills for adolescents to learn and practice in their life. The development of emotional maturation and agency among peer educators by expression of caring approaches toward their peers clarifies the ‘human capacity for response’ theory. The expression of human capacities toward the others has a positive impact on the capacity of the care giver. It is an expression of the personal and collective by which strengths are reflected, discovered, and expanded by a shared approach to addressing concerns.

### Implications and Suggestions for Further Research

Embedding these findings into a larger perspective, we identify a few important considerations. First, the outcomes of this study show that PE volunteers had an accelerated emotional maturation, accompanied by a sense of empowerment and agency. This is an important aspect to look at, particularly in relation to adverse effects that might arise from working with and listening to people in distress, such as ‘*fatigue’* or burn-out (Lewis & Manusov, 2009). It is suggested that the level of individual responsibility listeners feel can increase their own distress (Lewis & Manusov, 2009). It is therefore important to ensure that volunteers are made aware of the limits of their personal responsibility regarding anything that happens in the world around them; for them to deeply know that, while they may be concerned about it, they are not accountable for any suffering they witness. More importantly, the focus of any PE program should be on creating a notion of shared responsibility and working as a team. The current study indicates that the mutual bonding and sharing between PEs was well established in the PE network dynamics. Debriefing sessions that were conducted as a part of the program probably played an important role in facilitating sharing and bonding. We therefore suggest that PE programs can maintain the well-being and sustainability of volunteers, when debriefing sessions are incorporated as a part of the program.

Second, this study indicates that PE volunteers acted as listeners even outside the school boundaries. The subsequent skill development resulting from this involvement can open possibilities for their involvement in future programs on suicide prevention in higher educational institutions (such as universities), as well as in work placements. A panel study of high school seniors who were re-interviewed in their mid-20 s and again in their early 30 s Janoski, Musick, & Wilson (1998) show that volunteer work undertaken in high school has long-term influence on volunteering in middle age. Organizations that work in higher education spaces and involve volunteers can invite peer educators to participate in their programming. Thus, peer education programming can benefit both the peer educators and the students they support.

Third, and somewhat related to the second point, this study poses questions regarding the envisaged target group of PE programs. Adolescents undergo important social, cognitive, and emotional transitions that can at times cause major distress (Hussain et al., 2008). It is in such social turbulence that suicidal behavior, often impulsive, can arise (Dew et al., 2010), yet it is difficult to determine the individuals at risk. In our study, and through the observations/narratives of respondents, we found that, although none of the peer educators showed immediate suicidal tendencies, many of the distressing risk factors related to youth suicide in India (such as distress due to relational conflicts, academic stress, parental pressures, anxieties about self-image) (Radhakrishnan & Andrade, 2012) were also experienced by the peer educators. This is perhaps not surprising. It is reasonable to consider that people who show an interest in topics related to psychology are often dealing with psychological issues themselves, which they may hope to understand better through their participation in mental health work. Several studies, for instance, show that psychology students and practitioners experience high levels of anxiety, depression, and/or even suicidal ideation (Gilroy, Carroll, & Murra, 2002; Kleespies et al., 2011). Other research suggests that mental health workers are significantly more likely to have experienced psychological distress and dysfunctional dynamics in their childhood, including childhood trauma, than other professionals (Elliott & Guy, 1993). The participation in a PE program could therefore be particularly attractive to young adolescents who experience distress as it offers a means to understand themselves and provides a sense of purpose as they are beginning to explore and develop their own identity and value (Daniel & Goldston, 2009). PE programs should be developed in a way that helps students step out of a single-minded focus of mental burden, especially those who are at risk of developing suicidal tendencies (Shneidman, 1998), and for whom this PE program may present an answer; not as *receiving* help, but as active participant in the volunteering program. Daniel and Goldston (2009) therefore also suggest that *‘encouraging adolescents to participate in activities that involve helping other people (e.g., volunteerism) in an effort to help adolescents to gain perspective on their problems, develop their assets or strengths, and to foster “meaning’in their lives”’* (2009, p. 260). We feel that our study confirms that such purpose is given to the participating volunteers and might be a preventive and healing measure in itself for an otherwise concealed group of potential sufferers. Similarly, peer educators who are emotionally competent add to the reach and variety of the student support mechanism provided by schools. SALT-oriented peer education programs that enhance strengths are thus a good addition to extracurricular activities conducted as a part of the school programming.

Finally, this explorative study was not equipped to systematically measure ‘before and after’ changes in students against a control group. The study relied partly on self-reports, which for various reasons are likely to be somewhat biased. Nevertheless, the careful triangulation approach across different actors helped us distinguish which skills and changes in PE volunteers were highly likely to have been induced through their participation. Future research could take our findings further by introducing a baseline and include various psychometric scales to study outcomes related to emotional awareness and agency in particular. Similarly, further research could focus on substantiating these findings in a larger sample, in different schools, and in different geographical contexts, as well as in other thematic areas of peer education, such as HIV programs, or drug use, sexual health. In this way, schools can contribute to complex challenges in contemporary society, beyond the boundaries of the school as an educational institute.

## Appendix 1

See Tables 1 and 2.

## Appendix 2

See Table 3.

## Footnotes

1 Henceforth called ‘Connecting’.
